# Multiple environmental stressors mediate cyanobacteria recruitment in microcosms simulating spring conditions from two Midwest US hypereutrophic reservoirs

**DOI:** 10.1093/plankt/fbaf045

**Published:** 2025-09-09

**Authors:** Maggie Voyles, Lesley B Knoll

**Affiliations:** Department of Biology, Miami University, 700 E High St, Oxford, OH 45056, USA; Department of Biology, Miami University, 700 E High St, Oxford, OH 45056, USA

**Keywords:** resting cells, multiple stressors, experiment, microcosm, akinetes

## Abstract

Cyanobacteria harmful algal blooms (cyanoHABs) are a complex threat to water quality. Most research to date on the drivers of cyanoHABs focuses on environmental factors in the typical “growing season” despite evidence that cyanobacteria overwintering dynamics may have substantial effects on cyanobacteria seasonal succession and bloom formation. Additionally, the growing season is now beginning earlier and ending later in many parts of the world. Here, we examine the impacts of light, temperature and nutrients on the magnitude and timing of cyanobacteria recruitment from sediments in two hypereutrophic reservoirs in the Midwestern USA in the early spring season via microcosm recruitment experiments. We observed that recruitment was greatest at the first sampling point (Day 3), then declined throughout the rest of the 18-day experiment for both reservoirs. Further, increasing light and temperature significantly promoted recruitment in both systems, while nutrient additions were only a significant driver of recruitment in one lake. The recruited cyanobacteria community identity was similar in both lakes, with *Planktothrix*, *Raphidiopsis* and *Pseudanabaena* being most abundant. This study highlights the complex, interactive effects of environmental variables on cyanobacteria recruitment.

## INTRODUCTION

Cyanobacteria harmful algal blooms (cyanoHABs) are a threat to water quality in freshwaters because they can cause significant public health and recreation concerns through the production of toxins (Paerl and Huisman, 2008). Additionally, cyanoHABs can disrupt aquatic food webs because cyanobacteria are generally considered a low-quality food source for fish and zooplankton, and toxins produced by some genera can cause fish kills (Moustaka-Gouni and Sommer, 2020). Some freshwater systems in North America and Europe are experiencing increased cyanobacteria concentrations as climate and land use alterations create favorable conditions for cyanobacteria to outcompete other algal taxa (Carey *et al.,* 2012; Anneville *et al.,* 2015; Huisman *et al*., 2018; Smucker *et al.,* 2021; Taranu *et al.,* 2015). Further, the increase in frequency and intensity of harmful algal blooms coincides with significant modern climatic shifts. Harmful algal blooms are triggered by a myriad of climate and land use factors, including increased surface water temperatures, prolonged or stronger thermal stratification and high nutrient loading (Paerl, 1988; Smucker *et al.,* 2021). Global surface water temperatures are increasing (O’Reilly *et al.,* 2015) and causing an earlier onset of ice-off in temperate zones (Sharma *et al.,* 2021), leading to longer summers and altered stratification regimes (Smucker *et al.,* 2021; Woolway *et al.,* 2021, 2022). Extreme precipitation events (i.e. flooding and drought) are also occurring more frequently in the United States, increasing the amount of high and low streamflow occurrences (Naz *et al.,* 2018). Here, we examine the dynamics of spring cyanobacteria in two reservoirs in the Midwestern United States. A recent study indicates many Ohio reservoirs have experienced a steady increase in harmful algal blooms in recent decades possibly due to an earlier onset of increased surface water temperatures and thermal stratification, higher summer precipitation and elevated nitrogen concentrations (Smucker *et al.,* 2021).

**Fig. 1:**
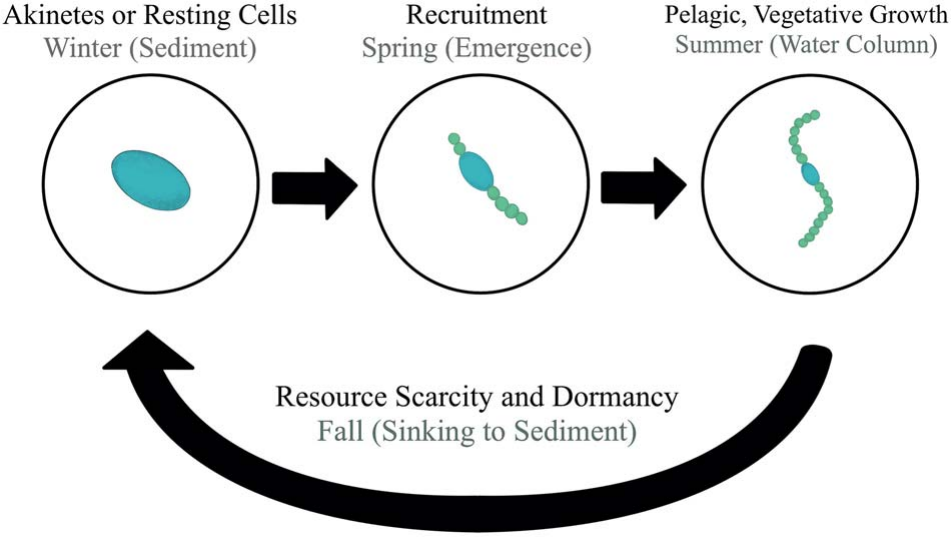
Conceptual diagram of the cyanobacteria life cycle.

Historically, most harmful algal bloom research has been centered around pelagic blooms in the summer months. However, cyanobacteria may remain active throughout the year (Ma *et al.,* 2016; Reinl *et al.,* 2023) or remain viable throughout the non-growing season by entering a dormant life stage (Fig. 1). This resting stage is thought to allow cyanobacteria to overwinter and withstand harsh environmental conditions (Reynolds, 1984; Cirés *et al.,* 2013) and may be a mechanism used to outcompete other algal taxa year-round (Carey *et al.,* 2012). Overwintering strategies vary among genera and include production of specialized akinetes, dormant resting cells or sustaining small populations in deep waters year-round (Reynolds, 1984; Kaplan-Levy *et al.,* 2010; Reinl *et al.,* 2023). Akinetes are specialized non-motile, spore-like cells that are rich in DNA and food reserves and are observed in heterocyst-forming genera like *Dolichospermum* (Kaplan-Levy *et al.,* 2010)*.* Resting cells, however, are dormant, often smaller vegetative colonies that are observed in *Microcystis* species among others (Reynolds, 1984). Akinete and resting cell development typically occurs after the peak bloom when light is limited from self-shading (Brunberg and Blomqvist, 2002; Kaplan-Levy *et al.,* 2010). These cells then accumulate in sediments and remain dormant until suitable environmental conditions are returned to. Some taxa, including *Planktothrix*, can persist in deep waters throughout the year due to their adaptive ability to survive in low light and temperature conditions, serving as an inocula for the following growing season (Babanazarova *et al.,* 2013; Reinl *et al.,* 2023). Additionally, *Planktothrix* may survive as shortened filaments called hormogonia during unfavorable climate conditions (Poulíčková *et al.,* 2004). Once required growth conditions are met, overwintering cyanobacteria (i.e. akinetes, resting cells and hormogonia) can germinate and migrate back into the water column—a process referred to as recruitment (Reynolds, 1984; Rengefors *et al.,* 2004). Overwintering cyanobacteria can remain dormant and viable in sediments for decades, which can cause substantial accumulation over time (Livingstone and Jaworski, 1980; Boström *et al.,* 1989).

Cyanobacteria recruitment may contribute to planktonic blooms. However, the significance of this contribution seems to be variable based on a limited number and scope of studies (Perakis *et al.,* 1996; Cottingham *et al.,* 2021). Estimates of the proportion of total planktonic cyanobacteria populations attributed to resting cell recruitment range from less than 1% to 60% in some cases (Hansson *et al.,* 1994; Barbiero and Welch 2006; Cirés *et al.,* 2013; Carey *et al.,* 2014). Additionally, the factors regulating both resting cell formation and germination are not fully understood. The current collection of knowledge suggests that cyanobacteria resting cell recruitment is dependent on spatial, temporal and environmental variables. Generally, recruitment is thought to be influenced most significantly by temperature, light, nutrients and sediment mixing (Ståhl-Delbanco *et al.,* 2003; Karlsson-Elfgren and Brunberg, 2004; Rengefors *et al.,* 2004), though the strength of effects of these variables may vary between genera (Kovács *et al.,* 2012). For example, recruitment in heterocyst-forming genera like *Dolichospermum* may be more limited by the availability of phosphorus than in non-heterocyst-forming genera (van Dok and Hart, 1997). Spatial variations in recruitment have also been indicated, as more recruitment is observed in littoral zone sediments because of the more favorable light and oxygen conditions for germination relative to that of pelagic zone sediments (Karlsson-Elfgren and Brunberg, 2004; Rengefors *et al.,* 2004; Ramm *et al.,* 2017). Lake bathymetry may also influence recruitment dynamics. Lakes with a large surface area of shallow sediments may enable cyanobacteria recruitment because of the increased light penetration and temperatures in bottom waters (Head *et al.,* 1999). Cyanobacteria recruitment has been shown to begin when temperatures rise and stratification begins in late spring to early summer (i.e. late May to early June) in northern temperate zones (Head *et al.,* 1999), but recruitment flux is highest in the summer (Verspagen *et al.,* 2005).

Water temperatures historically representative of spring and summer in the northern hemisphere are arriving earlier in the year due to increasing air temperatures (Woolway, 2023). Lake temperatures are increasing across the entire water column (Winslow *et al.,* 2015); reservoirs are becoming increasingly eutrophic due to anthropogenic nutrient sources like agriculture and urbanization (Khan and Mohammad, 2014); and stratification is prolonging and strengthening (Richardson *et al.,* 2017; Woolway and Merchant, 2019) in many systems worldwide. These changing spring and early summer (i.e. March through June) conditions may not only favor cyanobacteria over other taxa (Mooij *et al.,* 2005; Carey *et al.,* 2012; Kosten *et al.,* 2012), but may also shift the timing and increase the longevity of harmful algal blooms (Peeters *et al.,* 2007). With these changes occurring in the spring and summer in temperate zones, knowledge of the factors regulating cyanobacteria resting cell recruitment is critical to understanding harmful algal blooms holistically. Our research explores how changing spring climate conditions may influence cyanobacteria recruitment dynamics. In this study, we investigated how manipulating light, temperature and nutrients in two hypereutrophic reservoirs affects cyanobacteria recruitment in the spring. We predicted that cyanobacteria recruitment is an important process sustaining pelagic communities in these two systems and thus hypothesized that increased light availability, temperature and nutrient concentrations will promote cyanobacteria recruitment. Therefore, this research will provide important insight into the factors that may mediate cyanobacteria during the early spring season in hypereutrophic reservoirs.

### Study sites

**Fig. 2:**
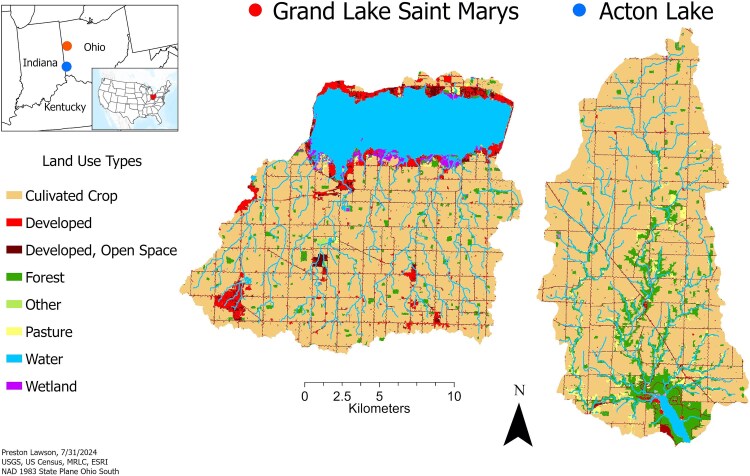
Land use map of the Acton Lake and Grand Lake St Marys watersheds.

We performed recruitment experiments on sediment collected from two hypereutrophic reservoirs in Ohio (USA), Acton Lake (39.573067, −84.749338) and Grand Lake St Marys (GLSM; 40.529429, −84.494345). While both the Acton Lake and GLSM watersheds are comprised of 80-90% agricultural land (Fig. 2), the Acton Lake watershed is dominated primarily by row crops (Vanni *et al.,* 2001), where significantly more animal agriculture is present in the GLSM watershed (Jacquemin *et al.,* 2018). Both Acton Lake and GLSM are relatively shallow. Acton Lake has an average depth of 3.9 meters and a surface area of 232 hectares (Vanni *et al.,* 2001), while GLSM has an average depth of 1.5 meters and a surface area of 5220 hectares (Jacquemin *et al.,* 2023). GLSM has been dominated by cyanobacteria like *Planktothrix* since 1970 due to decades of excess nutrient loading (Jacquemin *et al.,* 2023). Subsequently, GLSM frequently has toxin (microcystin) concentrations that exceed World Health Organization limits for safe recreation (Jacquemin *et al.,* 2023). While Acton Lake is dominated by cyanobacteria in mid to late summer, toxins rarely exceed advisory thresholds (Hayes and Vanni, 2018). Additionally, the species composition of cyanobacteria blooms in Acton Lake are historically variable depending on limiting nutrient identity (Hayes *et al.,* 2015). These two lakes are excellent sites for recruitment experiments because of their history of cyanobacteria blooms, the contrast in dominant taxa between sites and the high surface area of shallow sediments in both lakes.

## MATERIALS AND METHODS

**Fig. 3:**
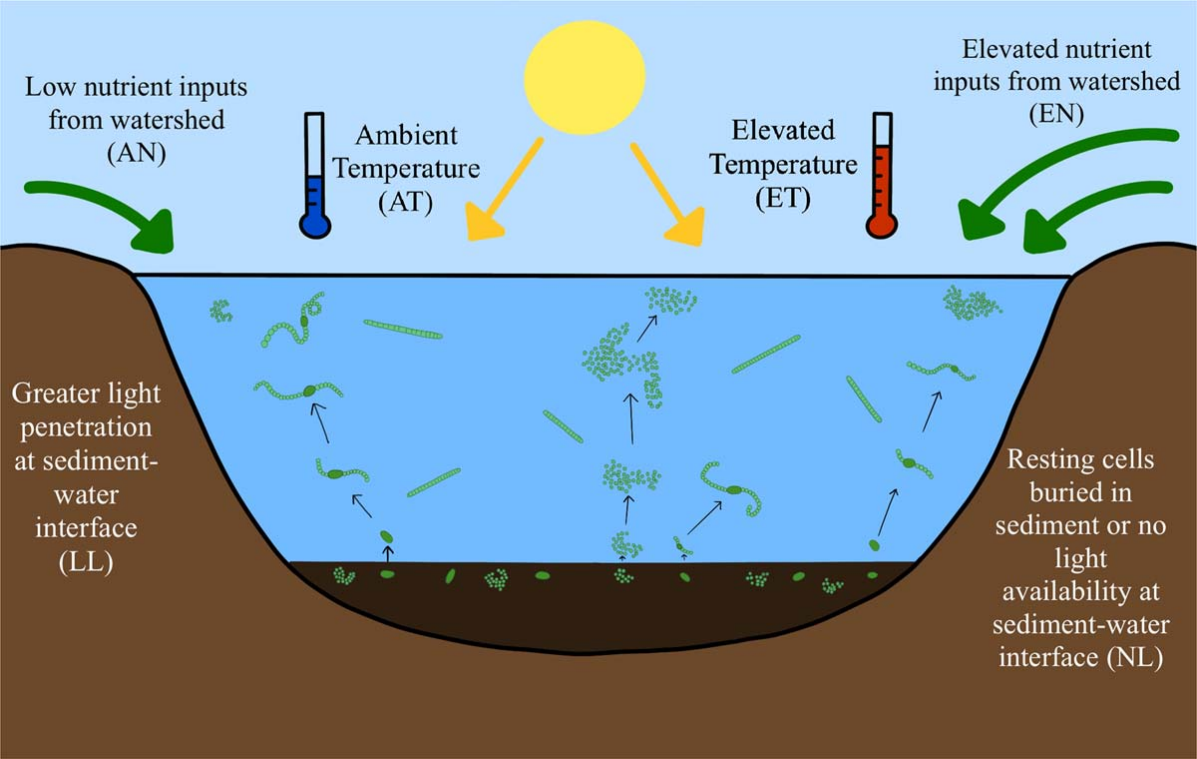
Simulated environmental conditions for the recruitment experiments. Light (no light & low light), temperature (ambient & elevated) and nutrients (ambient & elevated) were manipulated to understand the impacts on cyanobacteria recruitment.

We explored the effects of potential environmental drivers on cyanobacteria recruitment during the spring season using a 2 × 2 × 2 factorial recruitment experiment in which we manipulated light (no light, low light), temperature (ambient temperature, elevated by +3°C) and nutrients (N + P nutrient pulse, no nutrient pulse) (Fig. 3). Sediment samples were collected from 12 sites located in the littoral zones of both Acton Lake and Grand Lake St Marys using a gravity corer in March of 2024. The top two centimeters of surface sediment were preserved from each sample and pooled with all other sediment samples, resulting in one aggregated sample per lake. The temperature near the sediments upon collection was 8.2°C for Acton Lake and 7.3°C for Grant Lake St Marys. These samples were homogenized and stored at 4°C in the dark for 3 weeks prior to the recruitment experiments. This rest period was employed to either promote a shift into resting stages or kill any incidental vegetative filaments remaining in the small amount of water collected in sediment samples, thus contributing to the likelihood that the observed responses in this study were driven by recruitment and not pelagic growth (Rengefors *et al.,* 2004).

Following methods by Rengefors *et al.* (2004), we conducted microcosm recruitment experiments in 50 mL Pyrex glass test tubes, and each treatment had three replicates. For each lake, there were 24 experimental units (i.e. test tubes) for a total of 48 experimental units (3 replicates × 8 treatments × 2 lakes). 5 mL of homogenized sediment was added to the bottom of the tubes using a pipette with a wide tip. We sterilized water from each lake prior to the experiment by first filtering through 0.7 um Whatman GF/F filters, then through 0.22 um Sterivex filters using a peristaltic pump to remove plankton, bacteria and suspended solids but preserve ambient nutrient conditions. 40 mL of sterile lake water was carefully added to each tube using an angled syringe, ensuring minimal sediment resuspension. We placed the tubes in environmental chambers at either the average lake temperature in April 2023 in Acton Lake (12.5°C; Ambient Temperature, “AT”) or 15.5°C (Elevated Temperature, “ET”). We chose to increase the warming treatment by 3°C because the RCP 4.5 scenario for the Midwest USA predicts this approximate increase for parts of the region by ~ 2050 (Baker *et al.,* 2023). In each environmental chamber, half of the tubes were placed in a dark box to represent periods in which no light is available at the sediment–water interface in both experimental lakes (No Light, “NL”), and the other half was placed under grow lights at ~ 10 μmol PAR m^−2^ s^−1^ to simulate elevated light penetration (Low Light, “LL”) similar to conditions when light reaches the sediments in these productive lakes. Nutrient pulses mimicking a spring storm event in the Acton Lake watershed (80 μg/L P as NaH_2_PO_4_, 2.85 mg/L N as NaNO_3_ and 0.15 mg/L as NH_4_Cl) were added to half of the tubes (Elevated Nutrients, “EN”). The other half did not receive nutrient additions (Ambient Nutrients, “AN”). These concentrations reflect those found in a typical storm event in Acton Lake (Vanni *et al.,* 2001; Williamson, 2020), which we considered representative of other reservoirs in Ohio found in highly agricultural watersheds.

We sampled test tube microcosms every 3 days for 18 days. On each sampling day, all 40 mL of water in each tube was carefully removed using a pipette (Eppendorf Pipet Helper) without disturbing the sediment. 40 mL of sterile filtered lake water was then carefully replaced in each tube using an angled syringe to minimize sediment resuspension. Although this technique limited sediment resuspension, there may have been slight disturbance at the sediment–water interface. However, the same personnel replaced the water each time, helping to minimize variability in resuspension between tubes or days. 25 mL from each sample was immediately analyzed using an AlgaeLabAnalyzer (ALA; bbe moldaenke, Schwentinental, Germany) to determine the concentration (μg chlorophyll L^−1^) of chlorophytes, cyanobacteria, diatoms and cryptophytes. This 25 mL sample was then pooled with the remaining 15 mL that was not analyzed in the ALA sample in a 60 mL glass bottle for preservation for microscopy. The cuvette used for the ALA sample was then rinsed with 10 mL of Milli-Q and poured into the respective glass bottle between each treatment. Each sample for microscopy was preserved with Lugol’s iodine.

To provide context for the ALA trends, we also identified and enumerated phytoplankton to qualitatively explore general patterns of the dominant genera present in the experiments. For both lakes, only one replicate from each treatment was counted. For GLSM, samples from experiment Days 3, 6 and 12 were counted. These days were selected to include the first sampling point to confirm vegetative growth was not occurring (Day 3), a midpoint in which substantial recruitment was still observed (Day 6), and a day where little recruitment was observed (Day 12). For Acton, only samples from experiment Days 3 and 12 were counted due to a sampling error in Day 6 in Acton. Counts were performed at 400x on an inverted microscope (Zeiss model 473307-9902). The entirety of each preserved sample (50 mL) was poured into a 100 mL glass graduated cylinder and settled for at least 24 hours (Edler L., 1979; Paxinos and Mitchell, 2000). The top ~ 35 mL was then slowly siphoned using an angled syringe with a flexible plastic tube attached to the end. The remaining ~ 15 mL settled sample was then homogenized and placed into a small glass vial. 2.973 mL of each settled sample was placed into a HydroBios slide and allowed to resettle for at least 24 hours before being counted. For all slides, we standardized our counting procedure such that 20 independent fields of view were counted and identified to the finest taxonomic level possible, which was often the genus level. Phytoplankton were enumerated as natural units (i.e. filaments for filamentous, colonies for colonial and cells for single-celled). In some experimental samples, the total number of natural units counted was low because of limited recruitment. Thus, we focus on qualitative trends rather than quantitative analyses. All phytoplankton were enumerated, with the exception of phytoplankton around the picophytoplankton size range. Using an ocular micrometer, we measured up to 20 individuals per taxon based on the respective designated geometric shape (Hillebrand *et al.,* 1999), but in the case of taxa with fewer individuals, we measured all individuals encountered. Biovolume (μm^3^/mL) was then determined by multiplying the calculated volumes by the number of units counted per mL.

During April, the same month of the experiments, we also collected water from 0.5 m from each lake for dissolved nutrients (ammonium-N (NH_4_-N), nitrate/nitrite-N (NO_3_-N) and soluble reactive phosphorus) and total nutrient (total nitrogen, total phosphorus) concentrations. Dissolved nutrients were filtered through Pall A/E glass fiber filters (1 μm). Both total and dissolved nutrients were stored at 4°C after acidification with sulfuric acid to pH < 2 and were analyzed with a Lachet QC 8000 autoanalyzer.

### Data analysis

The effect of time on cyanobacteria recruitment was analyzed through repeated measures ANOVA using the “aov” function in R (version 4.4.1). Day 6 was omitted in Acton Lake analyses due to a sampling error. Multiway (3-way) ANOVA was then performed using the “aov” function in R to analyze the effects of each treatment on Day 3—the day in which the most recruitment was observed in both lakes as determined by ALA readings. Follow-up multiple comparisons for interaction terms from multi-way ANOVA were performed using the “emmeans” function in R (version 4.4.1).

## RESULTS

### Ambient lake nutrient concentrations

In Acton Lake, April nitrate and total nitrogen concentrations were higher (4200 μg N L^−1^, 5173 μg N L^−1^, respectively) than in Grand Lake Saint Marys (1020 μg N L^−1^, 2951 μg N L^−1^, respectively). April concentrations were all higher in Grand Lake Saint Marys than Acton for ammonium (290 μg N L^−1^, 189 μg N L^−1^, respectively), soluble reactive phosphorus (17.9 μg P L^−1^, 5.5 μg P L^−1^, respectively) and total phosphorus (221 μg P L^−1^, 139 μg P L^−1^, respectively).

### ALA: Cyanobacteria chlorophyll response

In both lakes, cyanobacteria were the dominant group observed in ALA readings across each sampling point and treatment. For both Acton Lake and GLSM the three most abundant cyanobacteria taxa observed were *Planktothrix aagardhii*, *Pseudanabaena* and *Raphidiopsis* (Table I, Figs. S1 and S2)*.* A qualitative description of the time dynamics of these taxa are in the supplemental material (Text S1).

**Table I:** Cyanobacteria taxa observed via microscopy with three dominant taxa bolded

Lake	Taxa Observed
Acton Lake	***Planktothrix, Pseudanabaena, Raphidiopsis,** Dolichospermum*
Grand Lake Saint Marys	***Planktothrix, Pseudanabaena, Raphidiopsis,** Dolichospermum, Cylindrospermopsis, Aphanazimenon*

**Fig. 4:**
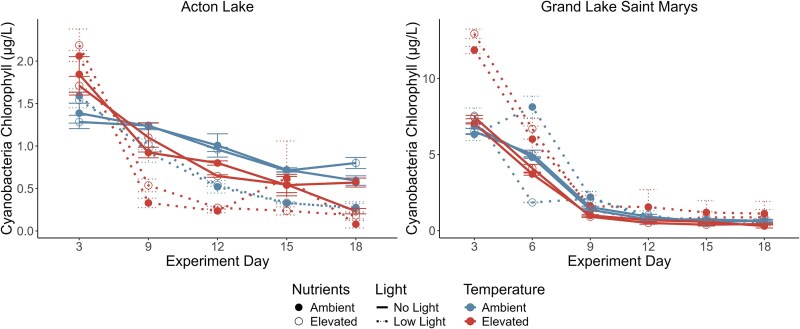
Cyanobacteria recruitment by treatment and experiment day in Acton Lake (left) and GLSM (right). The average value for all three replicates of each treatment is represented by points, while error bars visualize standard error. Closed circle points represent ambient nutrient treatments, and open circle points represent elevated nutrient treatments. Temperature is represented by color. Dashed lines represent low light treatments, whereas solid lines represent no light treatments. Repeated measures ANOVA showed a significant decline in recruitment over time following the peak day for both Acton Lake and GLSM (*P* < 0.01). Experiment Day 6 has been omitted for Acton Lake due to a sampling error.

We observed the most cyanobacteria recruitment on experiment Day 3 (our first sampling day) in both the Acton Lake and Grand Lake Saint Marys experiments (Fig. 4), with highest cyanobacteria chlorophyll concentrations of 2.187 μg/L and 12.94 μg/L respectively. Recruitment then steadily declined across all treatments in both lakes as the experiment progressed. Repeated measures ANOVA showed a significant effect of time on recruitment in both experiments (*P* < 0.01).

**Fig. 5:**
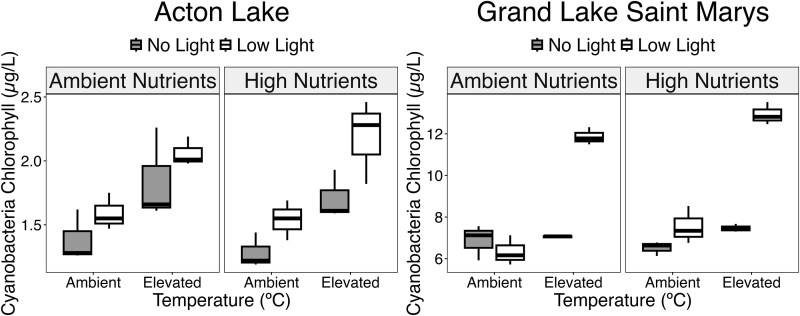
Cyanobacteria recruitment on experiment Day 3 in Acton Lake (left) and GLSM (right). Median cyanobacteria recruitment for each treatment is represented by the line inside each box. Upper and lower thresholds of boxes represent Q3 and Q1, respectively. Gray shaded boxes represent no light treatments, and open boxes represent low light treatments. No differences in recruitment were observed between nutrient levels in Acton (*P* = 0.66). Elevated light and temperature conditions independently promoted more cyanobacteria recruitment (*P* = 0.006 & *P* < 0.001, respectively) in Acton. A small difference was observed in cyanobacteria recruitment between nutrient levels in GLSM (*P* = 0.024). Recruitment in GLSM was promoted most when light and temperature were both elevated (*P* < 0.001).

For the peak day of observed recruitment in Acton Lake (Day 3), light and temperature additions both independently increased cyanobacteria concentration (μg/L chlorophyll) (Fig. 5). Three-way ANOVA for this day showed a significant effect of light (*P* = 0.006) and temperature (*P* < 0.001), but no significant nutrient effect (*P* = 0.665) and no interactions (Fig. 5; Table II). Multiple comparisons confirmed that elevated light and elevated temperature promoted recruitment on experiment Day 3 in Acton Lake.

**Table II:** Summary of statistical output from multi-way ANOVA for experiment Day 3 in Acton Lake and Grand Lake Saint Marys. Significant terms (P < 0.05) bolded

	Light	Temperature	Nutrients	Light × Temperature	Light × Nutrients	Temperature × Nutrients	Light × Temperature × Nutrients
*Acton Lake*							
p-value	**0.006**	**< 0.001**	0.665	0.529	0.340	0.691	0.576
f-value	10.14	30.49	0.195	0.415	0.748	0.164	0.326
df	16	16	16	16	16	16	16
*Grand Lake Saint Marys*							
p-value	**< 0.001**	**< 0.001**	**0.024**	**< 0.001**	**0.032**	0.513	0.352
f-value	130.91	163.63	6.17	107.91	5.56	0.446	0.918
df	16	16	16	16	16	16	16

For GLSM on the peak day, nutrient additions had a significant effect on recruitment (*P* = 0.024). Additionally, an interactive effect between temperature and light was observed (*P* < 0.001) (Fig. 5; Table II). Multiple comparisons for this interaction showed that recruitment was only increased when temperature and light were both elevated regardless of nutrient concentrations, while elevated light and elevated temperature conditions did not significantly increase recruitment independently (Fig. 5; Table II). A significant interaction between light and nutrients was also observed (*P* = 0.032). Multiple comparisons showed that recruitment was promoted more by light additions when nutrients were elevated compared to ambient (Fig. 5, Table II).

## DISCUSSION

### Light effects

Our study shows that cyanobacteria recruitment occurred in both systems and was influenced by environmental drivers. Light availability promoted recruitment in the experimental microcosms of both lakes. While recruitment occurred in both no light and low light treatments for both lakes, recruitment was higher across all other treatment combinations when light was available in Acton Lake sediments. In GLSM, however, light only significantly increased recruitment when temperature was also elevated. In both systems, we observed a steeper decline in recruitment after the peak day in the low light treatments relative to the no light treatments, suggesting that the cyanobacteria seed bank was exhausted more rapidly when light was available. The light effects we observed confirm the findings of previous recruitment experiments, highlighting the importance of light for akinete and resting cell germination (Rengefors *et al.,* 2004; Borges *et al.,* 2016). While light availability has historically been indicated as a requirement for recruitment due to the photosynthetic requirements of phytoplankton (van Dok and Hart, 1997), recruitment of some species has been observed in no light conditions when nutrients are abundant (Kaplan-Levy *et al.,* 2010). In our study systems, we observed that while light availability is optimal for cyanobacteria recruitment, it is not necessarily a prerequisite for the process. Since both Acton Lake and GLSM are hypereutrophic systems with low transparency, light is often limited at the water–sediment interface and is often unavailable during certain times of the year, particularly in deeper areas as is characteristic of hypereutrophic systems (Wetzel, 2001). This suggests that while cyanobacteria recruitment may continue to occur in these systems in the dark, it may be more substantial in the early spring and early summer before maximum water column shading from summer blooms has been reached.

### Temperature effects

Elevated temperature has been observed to promote recruitment of an array of cyanobacteria taxa in similar lab experiments (Rengefors *et al.,* 2004; Borges *et al.,* 2016) and field studies (Cao *et al.,* 2008). In Acton Lake microcosms, increased temperature independently promoted cyanobacteria recruitment. However, elevated temperature only significantly increased recruitment when light was available in GLSM microcosms. Thus, our study confirms the importance of temperature in regulating cyanobacteria recruitment. While the optimal temperature for cyanobacteria germination has been estimated to be as high as 35°C (Calomeni-Eck *et al.,* 2024), recruitment of some taxa has been observed in temperatures as low as 5°C (Cao *et al.,* 2008). Further, maximum germination rates have been observed at the optimal growth temperatures for pelagic vegetative cells of several cyanobacteria species, suggesting that temperature conditions may mediate species composition (Kaplan-Levy *et al.,* 2010; Calomeni-Eck *et al.,* 2024). Thus, while the rate of recruitment may be increased by elevated temperatures in Acton Lake and Grand Lake Saint Marys (Urrutia-Cordero *et al.,* 2020), recruitment may still occur in these systems during low temperature conditions (Holland and Walsby, 2008) and may favor species with low temperature thresholds like *Planktothrix* (Holland and Walsby, 2008). Particularly, recruitment of a dominant taxon, *Planktothrix,* may have been favored in this study by simulating spring conditions with relatively low temperatures.

### Nutrient effects

Nutrient additions have been shown to promote recruitment in many systems. However, the effect of additional nutrients is thought to be of decreasing significance as a system becomes more eutrophic (Rengefors *et al.,* 2004) as overwintering cyanobacteria may receive the necessary nutrient threshold for successful recruitment from the sediments in eutrophic and hypereutrophic systems (Park *et al.,* 2018). However, akinete germination generally incurs a heightened resource demand in contrast to resting cell germination due to the complex morphological and physiological changes that must occur for these cells to enter the pelagic life stage (van Dok and Hart, 1997; Kaplan-Levy *et al.,* 2010; Kovács *et al.,* 2012). In this study, we only observed a significant increase in recruitment with nutrient additions in GLSM microcosms. Maximum observed chlorophyll of recruited cyanobacteria varied between lakes with peak concentrations for Acton and GLSM at 2.187 μg/L and 12.94 μg/L respectively. It is not clear why GLSM cyanobacteria responded to nutrients while Acton cyanobacteria did not. Since *Raphidiopsis* (an akinete-forming genera) was generally the second most abundant taxon observed in GLSM by biovolume, but was only observed in low volumes in Acton Lake (Figs. S1 and S2), the promotion of growth of this taxon by nutrient additions could be a driver of the observed differences in effects of nutrients between lakes in this experiment. Further, the overall recruited cyanobacteria biomass in Acton Lake was low and the primary taxa observed overwinter as resting cells, the nutrient additions in these experiments may have been negligible in comparison to the nutrient-rich conditions in the sediments in Acton Lake (Nowlin *et al.,* 2005; Knoll *et al.,* 2014). However, we note that surface sediment phosphorus concentrations in Ohio reservoir sediments were lower than predicted for eutrophic lakes (Carey and Rydin, 2011). This may be a result of high terrestrial inputs in reservoirs compared to eutrophic natural lakes, with the latter possibly having a higher fraction of phytodetritus that is rich in phosphorus (Knoll *et al.,* 2014).

### Cyanobacteria community responses

While there were slight differences in community assemblages in Acton Lake and GLSM, the same three cyanobacteria taxa were dominant in both systems: *Planktothrix*, *Pseudanabaena* and *Raphidiopsis*, which collectively represent both resting cell and akinete overwintering strategies (Kaplan-Levy *et al.,* 2010; Reinl *et al.,* 2023). All three of these taxa are known to survive during unfavorable environmental conditions, including the low light (darkness & ~ 10 μmol PAR m^−2^ s^−1^) and temperature (12.5°C & 15.5°C) conditions simulated in this study (Holland and Walsby, 2008; Jia *et al.,* 2021; Reinl *et al.,* 2023). *Planktothrix* and *Pseudanabaena* can persist throughout the winter even during periods of ice cover due to their ability to outcompete other cyanobacteria and survive under temperature stress (Poulíčková *et al.,* 2004; Dokulil and Teubner, 2012; Babanazarova *et al.,* 2013; Reinl *et al.,* 2023). Additionally, *Raphidiopsis* can withstand a wide range of environmental conditions including temperatures as low as 10°C (Dokulil, 2016; Jia *et al.,* 2021), and can specifically outcompete other cyanobacteria when irradiance and phosphorus are limited (Kovács *et al.,* 2012). Further, *Planktothrix* has been observed to survive in low light conditions, including complete darkness (Reynolds *et al.,* 2002; Holland and Walsby, 2008). This shows that the typical environmental conditions in the early spring in northern temperate lakes may favor the recruitment of cyanobacteria that are particularly adapted to low temperature and light over taxa with higher temperature and light requirements.

### Study limitations and future considerations

While this study addresses multiple drivers of cyanobacteria recruitment in hypereutrophic systems, many more natural lake processes beyond the scope of this experiment may influence recruitment. Sediment mixing and bioturbation were not simulated in this study but have been indicated as important mediating factors for cyanobacteria recruitment (Karlsson-Elfgren *et al.,* 2004; Rengefors *et al.,* 2004; Carey *et al.,* 2014). Particularly in shallow reservoirs like those in this study, turnover and resuspension events can occur frequently, and many similar systems support a large population of bottom feeding fish like gizzard shad (*Dorosoma cepedianum*) (Vanni *et al.,* 2005). This indicates that sediment disturbance is likely an important driver of *in situ* recruitment dynamics and should therefore be considered when discussing realistic recruitment scenarios. Further, many regularly occurring ecosystem processes like micronutrient fluxes, light periodicity and stratification patterns may influence recruitment but were not explored here. Thus, we are unable to isolate and comprehensively characterize the effects of the stressors manipulated in this study in a real lake ecosystem under changing climate conditions without considering the myriad biotic and abiotic interactions that are inherently lost in microcosm experiments. Furthermore, although our study design allowed us to test the influence of various factors on recruitment, we used small test tubes (50 mL) with only a small quantity of lake sediments. Thus, our findings from the microcosm experiment may not scale reliably to the whole lake. We suggest that future studies consider using other approaches to test these questions such as using intact cores for experimental manipulations, deploying recruitment traps in a gradient of lakes or using field-deployed experiments (e.g. limnocorrals, mesocosms).

## CONCLUSION

As cyanoHABs become an increasingly common threat in the temperate Midwestern US reservoirs (Smucker *et al.,* 2021), understanding the factors influencing the underappreciated process of cyanobacteria recruitment is essential. This study further displays that cyanobacteria recruitment is influenced by multiple, interacting environmental stressors and subsequently may be impacted by changing spring conditions in this region. Particularly, in the hypereutrophic reservoir study systems explored here, elevated light penetration and elevated temperature increased cyanobacteria recruitment. Additionally, alternative to our hypothesis, nutrient additions did not significantly affect recruitment in Acton Lake, suggesting that nutrient requirements of resting cells may be met by nutrient concentrations in the sediments of some hypereutrophic reservoirs. Thus, our study highlights the complex and sometimes opposing effects of environmental conditions on cyanobacteria recruitment.

## Supplementary Material

Supplemental_material_fbaf045

## Data Availability

Original data for this project are available via Scholarly Commons at Miami University: http://hdl.handle.net/2374.MIA/7050.
