# Supplementary material for: Multiple environmental stressors mediate cyanobacteria recruitment in microcosms simulating spring conditions from two Midwest US hypereutrophic reservoirs
Source: J Plankton Res. 2025 Sep 9;47(5):fbaf045. doi: 10.1093/plankt/fbaf045 (PMC12418944; doi:10.1093/plankt/fbaf045)
Supplement: Supplemental_material_fbaf045 [file supplemental_material_fbaf045.docx]

***Supplement to*:** Multiple environmental stressors mediate cyanobacteria recruitment in microcosms simulating spring conditions from two Midwest US hypereutrophic reservoirs

Maggie Voyles^1^, Lesley B. Knoll^1^

^1^Department of Biology, Miami University, 700 E High St, Oxford, OH, USA 45056

**Number of Figures**: 2

**Number of Text**: 1


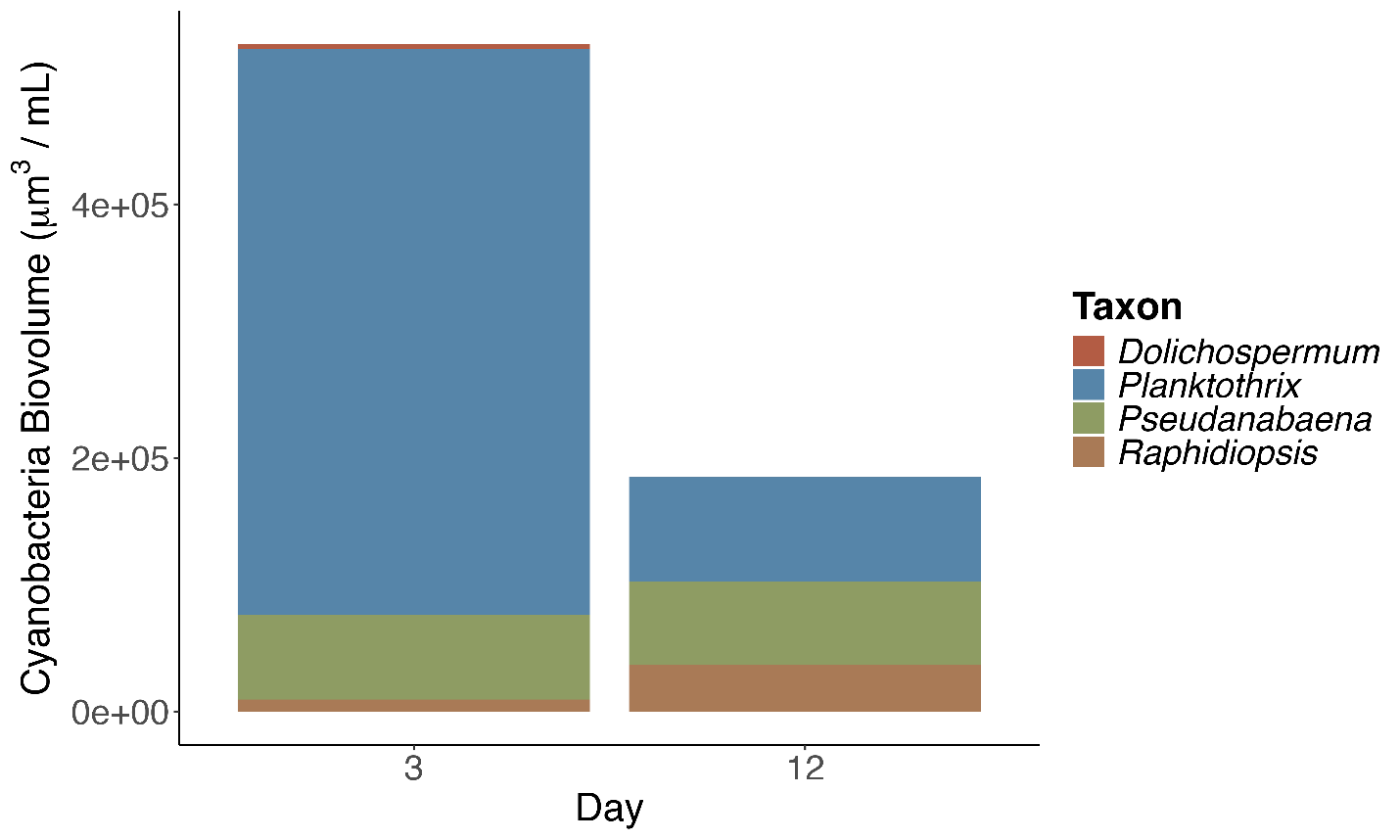


**Figure S1**. Mean cyanobacteria biovolume across the eight treatments in Acton Lake recruitment experiments on days 3 and 12.


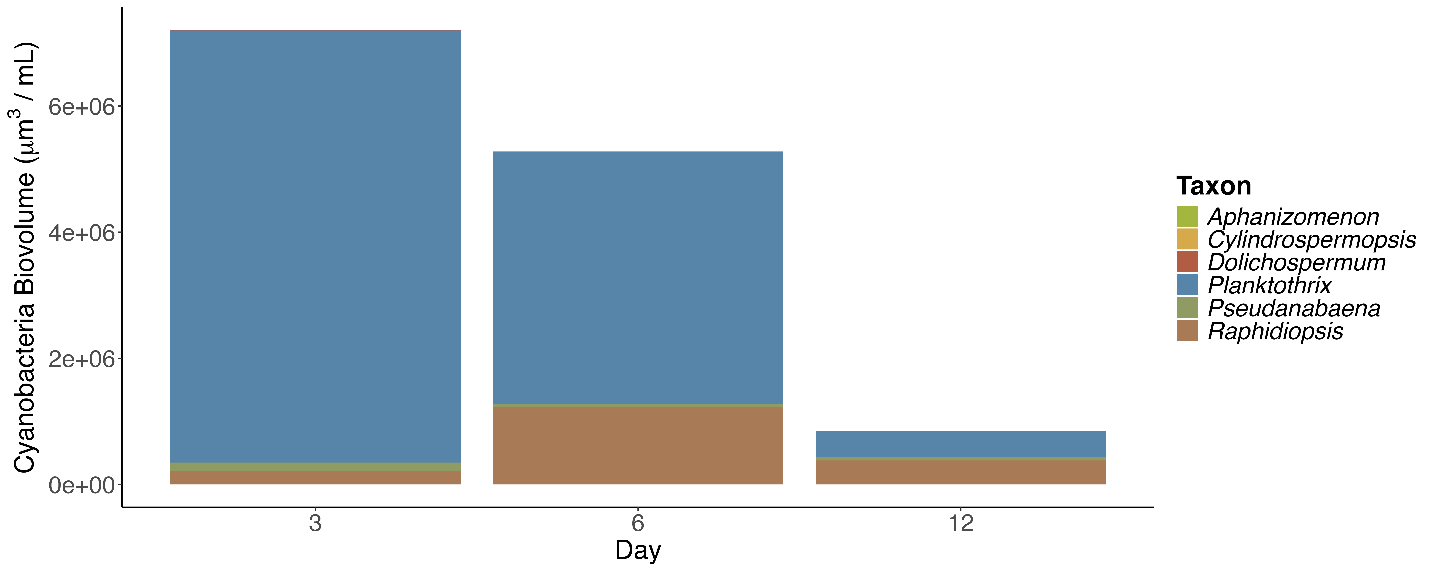


**Figure S2**. Mean cyanobacteria biovolume across the eight treatments in Grand Lake St Marys recruitment experiments on days 3, 6, and 12.

Text S1: Qualitative description of cyanobacteria community responses

Across treatments in Acton Lake on experiment day 3, *Planktothrix* was the dominant taxon by biovolume (Fig. S1). On day 12 in Acton Lake, *Planktothrix* biovolume decreased, but *Pseudanabaena* biovolume remained relatively constant between days (Fig. S1). In Acton Lake, *Raphidiopsis* biovolume was low on day 3, but increased on day 12 across treatments (Fig. S1).

Across treatments in Grand Lake St Marys, *Planktothrix* biovolume was high on experiment day 3 and decreased on subsequent experiment days (Fig. S2). *Pseudanabaena* biovolumes remained relatively low across all experiment days in Grand Lake St Marys (Fig. S2). *Raphidiopsis* density was highest on day 6 in Grand Lake St Marys (Fig. S2).
